# Longer-term consequences of increased body checking in women at risk for eating disorders–a naturalistic experimental online study

**DOI:** 10.1371/journal.pone.0316190

**Published:** 2024-12-26

**Authors:** Gina Geiger, Vanessa Opladen, Maj-Britt Vivell, Silja Vocks, Andrea S. Hartmann

**Affiliations:** 1 Clinical Psychology and Psychotherapy of Childhood and Adolescence, Department of Psychology, University of Konstanz, Konstanz, Germany; 2 Clinical Psychology and Psychotherapy, Institute of Psychology, Osnabrück University, Osnabrück, Germany; Bangor University, UNITED KINGDOM OF GREAT BRITAIN AND NORTHERN IRELAND

## Abstract

Body checking is a common behavior in both the general population and individuals with body image disturbances. Cognitive-behavioral theories postulate that body checking reduces negative emotions in the short term, but over time contributes to the development and maintenance of eating disorder pathology. So far, few experimental studies have assessed these longer-term consequences, mostly under laboratory conditions, yielding inconsistent findings, and without considering individual vulnerability and specific personality traits. In a naturalistic experimental cross-over design, women with low (*n* = 76) vs. high (*n* = 103) body concern completed an online survey on trait characteristics (e.g., intolerance of uncertainty). After a two-day baseline to assess the daily amount of habitual body checking, participants underwent two three-day experimental conditions in randomized order, in which they were asked to exhibit typical vs. threefold increased body checking. Before and after conditions, participants completed state measures of eating disorder symptoms, body dissatisfaction, affect, and general pathology online. In women with high body concern, body image-related symptoms (i.e., drive for thinness, body dissatisfaction) and negative affect worsened in the increased body checking condition, whereas in the typical body checking condition, positive affect increased and no negative impact emerged. Conversely, women with low body concern remained unaffected, except for higher drive for thinness following the increased condition. Bulimic and depressive symptoms did not change in either group. The inclusion of intolerance of uncertainty from an exploratory perspective generally did not impact the results. Our findings regarding the high-risk group underscore the potential etiological relevance of body checking for body image disturbances and eating disorders. For individuals at risk and those already affected by eating disorders, it seems important to address individual body checking as early as possible within psychoeducation to prevent a presumably harmful increase in this behavior. Personality factors influencing vulnerability to body checking need to be further examined.

## Introduction

*Body checking* (BC) refers to repeated behaviors aimed at gaining information about one’s body shape, size, or weight (e.g., [1]). BC behaviors such as examining one’s physique in the mirror or comparing it to that of other people, testing the fit of one’s clothing, or weighing oneself are common in everyday life [2, 3] and may therefore initially be regarded as harmless. However, BC behaviors represent a prominent clinical feature [4], assumed to maintain eating disorders (EDs) [5] and positively associated with psychopathology, body dissatisfaction, and dietary restraint in ED samples (for a meta-analytical review: [6]). While compared to clinical samples, healthy individuals perform BC less frequently (e.g., [7]) and might differ in terms of the underlying cognitions [8] and motivations [5], associations with psychopathology have likewise been reported in non-clinical samples [1, 9]. Accordingly, BC is also discussed as a factor promoting the development of EDs [10]. Since body image disturbances are considered as the core psychopathology from which ED symptoms derive on a secondary basis [11], BC research should pay particular attention to the subthreshold population who show body-related concerns and are thus at risk of EDs.

Aiming to understand the development of full-blown ED pathology, cognitive-behavioral theories of EDs [2, 12] propose that individuals with certain psychological risk factors (e.g., fear of fatness, overconcern with body shape, internalized body ideals) respond differently to external (e.g., body- or food-related) stimuli. Due to their dysfunctional body self-schema, individuals with EDs have cognitive biases that are easily activated (i.e., selective attention, memory and interpretation, overestimation of body size, and extreme drive for thinness; reviewed in [12]). This activation results in increased negative emotions, which in turn promote biased information processing. For the purpose of quick emotional regulation, and typically due to a lack of more adaptive coping skills [13], individuals perform compensatory and other regulatory behaviors [14], such as BC or body avoidance, restrictive eating, compulsive exercise, self-induced vomiting, and laxative abuse. These behaviors are assumed to initially reduce negative emotions but to lead to a vicious cycle of negatively reinforced behavior and increased negative affect in the long term. Cognitive biases are confirmed and the individual’s belief in the utility of and need for the behavior is strengthened. In sum, according to the theories, BC behavior is maintained or increased, psychopathology is consolidated, and negative affect increases (e.g., [2]).

There is also empirical evidence of an association of these mechanisms with BC in non-clinical individuals. In line with the theories, a meta-analysis found a strong association between BC and ED symptoms among non-clinical female samples (*r* = .60, *p* < .001; [6]). Moreover, a correlational study showed that BC behaviors and cognitions significantly predicted engagement in typical ED-associated behaviors such as bingeing, purging, restraint eating, and compulsive exercise in a female university sample [10]. Additionally, the frequency of BC was found to predict impairment in mental quality of life in cross-sectional data from a female community sample [15].

Besides these associations found in non-clinical samples, several observational BC studies have reported similar findings in at-risk samples. Targeting the vulnerable population of non-clinical women with high body concern, Stefano et al. [16] used ecological momentary assessment to randomly assess participants throughout the day, and confirmed BC as a significant predictor of body dissatisfaction and negative affect. In a study by Farrell et al. [17], women’s mirror BC behaviors were examined on the basis of questionnaire answers. The results revealed that high compared to low body concern was significantly associated with a focus on disliked body parts, providing evidence for selective attention. Furthermore, when looking in the mirror, women with high body concern also reported experiencing more negative cognitions and emotions [17]. Two more studies retrospectively examined the course of emotional states around a remembered BC episode [7, 18]. In non-clinical women with high levels of body concern, negative affect significantly decreased from before to 15 minutes after the remembered episode, leading the authors to conclude that BC might actually downregulate negative emotions.

With regard to the experimental evaluation of the aforementioned cognitive-behavioral theories and empirical associations, study findings likewise draw an inconsistent picture, necessitating a careful consideration of the applied time frames of the effects reported in different studies. We adopt Opladen et al.’s definitions [19] and distinguish immediate short-term effects, which are directly attributable to one specific BC episode (e.g., [18]), from temporally unlimited long-term consequences of habitually performed BC (e.g., [9]). The present study addresses a middle category, i.e., cumulative longer-term consequences of multiple, repeated episodes within a defined time frame, which has only been investigated in a smaller number of studies: Shafran et al. [20] found that 30 minutes of critical BC, as compared to a neutral whole-body examination, led to significant but short-lived increases in body dissatisfaction, feelings of fatness, and body-related self-critical thinking in non-clinical women. Examining the cumulative impact of repeated BC on one day, Bailey and Waller [21] asked non-clinical women to check their wrist size every 15 minutes for eight hours in a more naturalistic setting. At the end of the day, general disordered eating attitudes and body dissatisfaction had not changed, but the fear of uncontrollable weight gain had significantly increased. This effect was more pronounced in women with inherently more negative eating attitudes, again highlighting the vulnerability of this risk group. Walker et al. [22] conducted four consecutive ten-minute laboratory sessions of critical BC in women with high body concern. Directly after the manipulation, body satisfaction was significantly lower, and in line with expectation, dissatisfaction with body parts and negative affect had increased. However, the results of the analysis of change over time contradicted the theory regarding the posited directions of effect, as body satisfaction, dissatisfaction with body parts, and negative affect in women with high body concern significantly improved from the final laboratory session to a one-week follow-up. Thus, there were no negative longer-term consequences for either condition, although the rather artificial laboratory BC procedures may function differently from multiple brief in vivo episodes conducted over time [22]. Using a more naturalistic experimental design, Opladen et al. [19] investigated the impact of three days of BC with either typical or increased frequency in non-clinical women. A comparison of pre-and post-states revealed a significant increase in negative affect in both conditions, suggesting that BC generally produces negative emotions over time, in line with theory. By contrast, anxiety, as a facet of negative affect, decreased, suggesting that BC may have exerted an anxiolytic effect in this sample. Contrary to expectation, there was no effect on ED symptoms in either condition. General pathology worsened in the increased BC condition but improved in the typical BC condition, possibly indicating that BC is innocuous among individuals without EDs [19].

In sum, BC is a common behavior that is postulated to promote the development and maintenance of EDs. However, the available research on BC shows notable inconsistencies, even across studies examining the same time frames of effects (i.e., short-, longer-, long-term), and experimental studies are lacking. The inconsistent findings might be due to interindividual differences such as specific personality traits. In particular, intolerance of uncertainty, which is defined as the tendency to react negatively to uncertain situations on an emotional, cognitive, and behavioral level [23], could play an important role as it has shown transdiagnostic relevance and is particularly prominent in EDs [24]. Among ED patients, research has found positive associations of intolerance of uncertainty with drive for thinness, body dissatisfaction, and negative affect [24], and indirect effects on emotional symptoms and compensatory ED behavior (i.e., restriction and purging; [25]). Given that attainment of certainty was found to be one of the most important functions determining why BC is conducted, both in a clinical [4] and a non-clinical sample [19], it seems likely that BC may serve as a safety behavior to reduce negative emotions and increase the sense of control, especially when trait intolerance of uncertainty is high [26].

The present study thus aimed to extend empirical knowledge about longer-term consequences of naturalistic BC in an experimental online setting and to explore the role of intolerance of uncertainty in BC with respect to the development of ED pathology. Therefore, we replicated Opladen et al.’s online study [19], and supplemented their design with the factor of vulnerability, by investigating non-clinical subsamples with low vs. high ED risk, i.e., low and high body concern. After undergoing a screening of body image concern and completing a basic survey, female participants were asked to record their mean amount of habitual BC over two days, which served as their personal baseline in our study. Following this, using a cross-sectional approach, we asked participants to carry out either this typical BC frequency or a threefold increased BC frequency, for three days each. All participants completed both conditions in randomized order, with a four-day washout break between the two conditions. State measures were captured using self-report before and after each three-day condition. Regarding our first objective to examine longer-term consequences of BC, we hypothesized that three days of BC would result in a deterioration of the dependent variables (i.e., ED pathology, body dissatisfaction, affect, and general pathology, *H1*), in line with Opladen et al. [19] and referring to the theoretical assumptions of Williamson et al. [12]. Moreover, we expected the effects to be stronger after the increased BC condition than after the typical BC condition (*H2*) and to be stronger in the high-risk group compared to the low-risk group (*H3*). Our second objective was to investigate the relationship between intolerance of uncertainty and differences in BC-induced consequences. In this regard, we expected a stronger negative correlation between our inverted measure of intolerance of uncertainty and baseline BC in the high-risk group than in the low-risk group (*H4*). Finally, we expected that in the increased BC condition, the extent of intolerance of uncertainty would explain a significant amount of variance in the change of the dependent variables from pre- to post-checking, beyond that of group membership (*H5*).

## Methods

### Sample

This study was coordinated from University of Konstanz in cooperation with Osnabrück University in Germany. It was approved by the ethics committee of the University of Osnabrück. Recruitment occurred in three waves from February 2022 until May 2023. Potential participants were recruited through advertisements in public places, university mailing lists, on social media, and via a portal for classified ads. Individuals were eligible to take part in the online screening if they identified as female, were aged between 18 and 65 years, had no current diagnosis of a mental disorder, and were not currently undergoing psychotherapeutic treatment. Exclusion criteria were acute suicidality, self-injurious behavior, or intoxication with psychotropic substances except for caffeine and nicotine. If participants completed all parts of the study, they were reimbursed with money, raffle entry, or study credits for students. An a priori power analysis using G*Power 3.1.9.4 [27] indicated that a total sample size of *N* = 179 was sufficient to detect medium effects with a power of .80. The choice of a medium effect size was based on the effects that previous comparable experimental studies have found (i.e., medium-to-large in the non-clinical sample [21]; small-to-medium in a sample with high vs. low body concern [22]) that led us to anticipate variability in the expected effect sizes. Since, additionally, a meta-analysis by Walker et al. [28] demonstrated moderate correlations between BC and negative affect, which is considered a key factor driving negative reinforcement in ED development [12], we decided to choose the middle way and aimed for medium effect sizes.

Of *N* = 313 individuals who passed the screening, *n* = 240 completed the study with all relevant data gathered within the predetermined time restrictions. To support data collection, participants who had forgotten to complete a survey or completed it too late were actively offered the chance to repeat the respective BC condition and questionnaires. As the naturalistic online design did not provide a great deal of control over participants’ actual implementation of the study requirements, we elaborated a thorough scheme for data cleaning (e.g., dealing with participant response behaviors, calculation of BC frequencies…), which is explained in S1 Fig. For the benefit of validity, *n* = 61 participants were reasonably excluded, as can also be seen in the participant flow in S1 Fig. The final sample consisted of *N* = 179 women, of whom *n* = 76 were screened as at low risk for EDs and *n* = 103 as at high risk.

### Procedure

On the landing page of the survey, participants were informed about the main goals of the study, confidentiality and privacy procedures, and the inclusion criteria, after which they provided consent to their participation in a short online screening questionnaire. First, a unique participant code was assigned for the purpose of anonymization and ability to link data sets. Following this, participants were asked to state their age, gender, and whether they were currently undergoing psychotherapy. Next, we used participants’ combined mean score on the Weight Concern and Shape Concern subscales of the Eating Disorder Examination-Questionnaire (EDE-Q; [29]) sensu Voges et al. [30] to assign them to either the low body concern/low ED risk group (combined mean score ≤ .38) or the high body concern/high ED risk group (combined mean score ≥ 3.36); individuals scoring in the mid-range (.38 < combined mean score < 3.36) were excluded. In the case of study admission, participants were taken directly to the basic survey. Before answering the questions, they received information about the requirements and the course of study and had to give consent to the privacy statement. Important terminology was explained, with additional examples to facilitate understanding (e.g., a BC episode was defined as every single behavior of BC, with a brief duration or lasting for minutes or hours). This was followed by questions about physical and mental health (height, weight, medication intake, physical illnesses, previous diagnosis/treatment of a mental disorder), and additional questionnaires that were not analyzed in the present study (see OSF preregistration). Finally, to facilitate the counting of performed BC episodes, participants were asked to install one of two recommended apps (iOS: *CountThatNow*, Android: *Thing Counter*).

**Fig 1:**
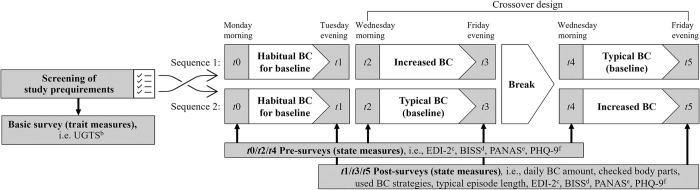
Procedure of study. ^a^Eating Disorder Examination-Questionnaire, ^b^Tolerance of Uncertainty Scale, ^c^Eating Disorder Inventory-2, ^d^Body Image States Scale, ^e^Positive and Negative Affect Schedule, ^f^Patient Health Questionnaire-9.

Each weekend, a new round of the experiment began, with participants receiving an email with preparatory instructions and an attached overview document providing guidance and answers to potential questions. Via an invitation link, participants could voluntarily and anonymously join a Telegram channel, which served to provide an additional reminder of the tasks of the current experimental condition and to reinforce motivation, while remaining anonymous among the other group members. It was further recommended that participants set alarms to remind them to complete the upcoming surveys. The official twelve-day data collection period (Fig 1) began on a Monday and required participants to track their daily amount of BC episodes on two normal consecutive days. The (rounded up) average across both days was set as their individual BC baseline. Over a three-day checking phase from Wednesday until Friday, participants were then either instructed to perform their typical BC frequency (typical BC condition) or to actively triple their BC baseline (increased BC condition) depending on the randomized sequence order. After a four-day washout phase, participants completed the respective other condition on the same weekdays. To assess various state measures before checking, surveys *t*0, *t*2, and *t*4 were sent via email at 6 am, and participants were asked to complete them after getting up (by 2 pm at the latest). Surveys *t*1, *t*3, and *t*5 were sent at 6 pm to capture participants’ post-checking state before going to bed (to be completed by 3 pm the next day at the latest). Post-surveys additionally determined whether instructions had been implemented, by asking about the number of BC episodes actually performed per day, the body parts checked, BC strategies used, and the length of a typical episode. Feasible time restrictions were implemented to accommodate our relatively young, primarily student participants, allowing them to complete the surveys within their varied daily routines. As we did not rely on questionnaire data from *t*0 or *t*1 for the analyses (except for the habitual amount of BC, which was re-captured at *t*2), the time frames of *t*1 and *t*2 were allowed to theoretically overlap, offering the advantage of familiarizing participants with the procedure. After completing each survey, participants were forwarded to an external webpage, where they entered their email address to ensure that participation occurred without privacy breach.

### Measures in the screening and basic survey

#### Eating Disorder Examination-Questionnaire (EDE-Q)

To assess ED pathology for the inclusion criterion of body concern, we used the German translation of the self-report questionnaire EDE-Q [31]. Respondents rate the incidence or intensity of 22 items (e.g., “Have you been deliberately trying to limit the amount of food you eat to influence your shape or weight (whether or not you have succeeded)?”) on a 7-point Likert scale from 0 = *no days*/*not at all* to 6 = *every day*/*markedly* referring to the last 28 days, with higher scores indicating more pronounced body concern. Internal consistencies of the German version were found to be overall high for the total score (*α* = .97) and subscale scores (.85 ≤ *α* ≤ .93; [32]) in individuals with EDs as well as subclinical, non-clinical, and control samples with mental disorders. In the present study, the two (out of four) subscales upon which group assignment was based both showed excellent internal consistency (*α*_Weight Concern_ = .93, *α*_Shape Concern_ = .97).

#### Tolerance of Uncertainty Scale (UGTS)

To consider the potential role of intolerance of uncertainty, we employed the German Tolerance of Uncertainty Scale (*Ungewissheitstoleranzskala* [UGTS; [33]). This self-report questionnaire comprises eight statements about uncertain situations (e.g., “I like trying things out, even if it doesn’t always lead to something”), which are rated on a 6-point Likert scale from 1 = *strongly disagree* to 6 = *strongly agree*. A high total score reflects high tolerance of uncertainty; this needs to be taken into account when interpreting our results, as we theoretically targeted and refer to intolerance (rather than tolerance) of uncertainty. Investigations with various samples yielded a questionable to acceptable internal consistency (*α* = .66–.72; [33]), which was exceeded in the present study (*α* = .78).

### State measures at *t*0–*t*5

The following questionnaires were administered before and after every checking phase. To increase sensitivity to change across BC conditions, the original time frames to which responses should refer were shortened to the past three days (with the exception of the Body Image States Scale [BISS; 34]). For the calculation of internal inconsistencies, Cronbach’s *α*_Ø_ was averaged across all time points from *t*2 to *t*5.

#### Eating Disorder Inventory-2 (EDI-2)

Expected BC-induced changes in ED symptomology were captured using two subscales from the EDI-2 ([35]; German version: [36]), i.e., *Drive for Thinness*, which refers to an excessive preoccupation with dieting and weight gain, and *Bulimia*, referring to a tendency towards uncontrollable overeating. For each subscale, participants rate the frequency of seven items (e.g., Drive for Thinness: “I am terrified of gaining weight”; Bulimia: “I eat when I am upset”) from 1 = *never* to 6 = *always*, with higher scores reflecting higher psychopathology. The EDI-2 subscales have shown good internal consistencies in a patient sample (*α*_Drive for Thinness_ = .85, *α*_Bulimia_ = .90) and a female control sample (*α*_Drive for Thinness_ = .86, *α*_Bulimia_ = .72; [37]); internal consistencies in the present study were even higher (*α*_ØDrive for Thinness_ = .94, *α*_ØBulimia_ = .92).

#### Body Image States Scale (BISS)

The BISS ([34]; German version: [38]) measures the current cognitive-affective body experience (e.g., regarding height, weight, shape, appearance, attractiveness) with six items rated on a 9-point Likert scale (e.g., “Right now I feel [Extremely dissatisfied to Extremely satisfied] with my physical appearance”). Our recoding differed from the original version, such that higher scores indicated higher body dissatisfaction. The scale showed excellent internal consistencies in the present study (*α*_Ø_ = .95), which were even higher than in Tanck et al.’s female sample (*α* = .82–90; [1]).

#### Positive and Negative Affect Schedule (PANAS)

The PANAS ([39]; German version: [40]) assesses positive and negative affect with two separate subscales, each comprising ten emotional adjectives (e.g., “enthusiastic” or “afraid”), with participants rating the degree to which they experience these emotions a 5-point Likert scale from 1 = *not at all* to 5 = *extremely*. Higher scores indicate more pronounced positive or negative affect, respectively. Due to a programming error, the first recruitment cohort could answer the PANAS with an additional false option of 6 = *strong*. As this semantically overlaps with the option of “extremely”, we converted item responses of 6 into 5 to reestablish the original scale. A comparison of the pre-checking affect in cohorts 1 (with the false scale) vs. 2 and 3 (with the correct scale) yielded no differences in pre-NA, but did reveal differences in pre-PA at *t*2 and *t*4. Therefore, we additionally present a reanalysis of PA data from cohorts 2 and 3 in the supplementary material. Good internal consistency has been demonstrated for both scales (*α*_Positive Affect/Negative Affect_ = .86; [40]); internal consistencies in the present study were excellent (*α*_ØPositive Affect_ = .93, *α*_ØNegative Affect_ = .90).

#### Patient Health Questionnaire-9 (PHQ-9)

The PHQ-9 is a brief depression severity measure ([41]; German version: [42]), which was used in the present study as an indicator of general pathology. Respondents rate how often they experience nine diagnostic symptoms (e.g., “little interest or pleasure in doing things”) on a 4-point Likert scale ranging from 0 = *not at all* to 3 = *nearly every day*, with higher scores indicating higher psychopathology. A good internal consistency of the PHQ-9 was reported in a primary care sample (*α* = .89; [43]) and confirmed in the present study (*α*_Ø_ = .86).

### Data analysis

All processing of data was conducted using the statistical software R 4.2.3 [44], its graphical interface RStudio 2023.6.0.421 [45], and the packages cited in S1 Appendix. The significance level was set at *α* = .05 for all analyses. Effect sizes of simple comparisons were based on Cohen’s *d* (small: .20, medium: .50, large: .80; [46]). As recommended by Bakeman [47] and Lakens [48], *η*^2^_G_ was reported for ANOVAs (small: .02, medium: .13, large: .26; [49]).

First, we calculated the mean number of BC episodes performed per day across the baseline phases and the two condition phases. The BC increase factor was calculated as the ratio of increased to typical BC. Next, descriptive statistics (*N*, *M*, *SD*, range) were computed for the final sample and the two subsamples. Group differences were examined using independent (Welch) *t*-tests. Analyses with dependent variables were always based on their mean scores at time points *t*2-*t*5, although sum scores were formed for the EDI-2 and PHQ-9, in line with convention.

To verify whether the instructions were followed correctly and the randomization was successful, we conducted the following manipulation checks: equivalence between baseline and mean typical BC frequency (*MC1*: paired Welch *t*-test); difference between conditions regarding their mean BC frequencies (*MC2*: one-sided paired Welch t-test); equivalence of BC increase factor between groups and sequences (*MC3*: 2×2 ANOVA); no sequence effects on mean BC frequencies in the different conditions (*MC4*: paired t-tests) or on dependent variables (*MC5*: 2×2×2×2 ANOVA with between-factor sequence).

To test *H1*-*3*, we ran six separate 2×2×2 mixed ANOVAs with the between-factor group (low vs. high ED risk) and the two within-factors condition (increased vs. typical BC) and time (pre vs. post), using the *anova_test()* function [50]. As assumptions of normal distribution and homogeneity of variance were violated, and the robustness of ANOVA does not apply to unequal sample sizes [51], correction for unbalanced data (type = 3) was applied. All significant main and interaction effects were followed up by an analysis of simple main effects and pairwise comparison with Bonferroni correction to avoid type I errors which is indicated with the adjusted p-value (*p*_adj_).

Mean scores of intolerance of uncertainty were not normally distributed in our sample, but were homoscedastic. Accordingly, *H4* was examined using Spearman correlation with Fisher’s z transformation [52] and Zou’s confidence interval [53] difference values. To assess *H5* for each dependent variable, we always compared two nested linear regression models, drawing on group status only or adding intolerance of uncertainty as a second predictor.

## Results

### Participant characteristics and group differences

Detailed information and group comparisons regarding participant characteristics are provided in Table 1. The mean age of the final sample (*M* = 23.1, *SD* = 5.5, range: 18–64) did not significantly differ between the two groups. Overall, *n* = 41 participants reported undergoing psychotherapy in the past. Only *n* = 8 women stated a previous ED diagnosis, of whom *n* = 5 were screened into the high-risk group. As intended, the EDE-Q score differed significantly between groups. While both groups were in the healthy weight range, the high-risk group had a significantly higher mean BMI than the low-risk group. Moreover, the high-risk group showed more frequent BC behavior at baseline and in the manipulated BC conditions, and the low-risk group showed significantly higher scores on our inverted measure of intolerance of uncertainty (i.e., greater tolerance).

**Table 1:** Descriptive statistics and group comparisons of participant characteristics.

	Low-risk group (*n* = 76)			High-risk group (*n* = 103)			Statistics
	*M*	*SD*	Range	*M*	*SD*	Range	Two-sided (Welch) t-test, Cohen’s *d*
Age	23.9	7.4	18–64	22.4	3.4	18–34	*t*(98.91) = 1.63, *p* = .11
BMI	21	2.3	17.4–27.9	24.9	5	16.0–50.5	*t*(151.33) = –6.89, *p* < .001, |*d*| = .99
EDE-Q score^a^	0.2	0.1	0–0.3	4.3	0.7	3.4–5.9	*t*(109.51) = –58.28, *p* < .001, |*d*| = 8.16
UGTS score^b^	3.4	0.8	1.4–4.8	3.2	0.8	1.8–5.1	*t*(177) = 2.47, *p* = .02, |*d*| = .37
BC baseline^c^	4.3	3.4	1–22	11	7.7	1–42	*t*(148.35) = –7.91, *p* < .001, |*d*| = 1.13
Mean typical BC^d^	4.4	3.1	1–18	10.7	7.6	1–45	*t*(144.34) = –7.66, *p* < .001, |*d*| = 1.09
Mean increased BC^d^	12.1	9.7	2.7–62.7	29.8	20.6	3–112	*t*(153.54) = –7.67, *p* < .001, |*d*| = 1.10
BC increase factor^e^	2.8	0.6	1.5–4.3	2.9	0.6	1.5–4.6	*t*(177) = –.56, *p* = .58

^a^EDE-Q score was built as the combined mean score on the subscales Weight Concern and Shape Concern in line with Voges et al. [30]. ^b^Higher scores on the UGTS indicate more tolerance of uncertainty (i.e., less intolerance). ^c^Baseline = the rounded average of the number of checking episodes on Monday and Tuesday, and should indicate the typical BC frequency. ^d^Mean BC frequency within conditions was averaged over three days of either typical or increased checking. ^e^BC increase factor = ratio of increased to typical BC (should be 3.0).

### Manipulation checks

*MC1* revealed no differences between the baseline and mean BC frequency of the typical BC condition (*t*(178) = .99, *p* = .32). Regarding *MC2*, the mean BC frequencies differed significantly between the typical and the increased BC condition (*t*(178) = 15.28, *p* < .001). In *MC3*, the mean BC increase factor (*M* = 2.83, *SD* = .60, range: 1.5–4.6) approached the intended threefold increase. It was not influenced by group (*F*(1, 175) = .53, *p* = .47), indicating equal commitment, but was influenced by sequence (*F*(1, 175) = 8.04, *p =* .01, *η*^2^_G_ = .044). In the Bonferroni-corrected follow-up, this effect of the order of conditions was only significant for the high-risk group (*p*_adj_ = .004). Successful randomization was proven by *MC4*, which showed that BC frequency was not influenced by sequence in the typical BC condition (*t*(177) = 1.02, *p* = .31) or in the increased BC condition (*t*(177) = 0.48, *p* = .63). *MC5* revealed no main or interaction effects of sequence on the dependent variables drive for thinness, bulimia, positive affect, and general pathology. However, sequence affected body dissatisfaction through a barely significant fourfold interaction (*F*(1, 175) = 3.91, *p* = .049, *η*^2^_G_ = .002). The follow-up analysis revealed a significant difference between conditions only in the high-risk group at post-measurement when the increased BC condition was conducted first (S1: *p*_adj_ = .02). Moreover, negative affect was influenced by a significant interaction between sequence and time (*F*(1, 175) = 5.5, *p* = .02, *η*^2^_G_ = .002), which disappeared in the follow-up (pre: *p*_adj_ = .20, post: *p*_adj_ = 1.00). In summary, the critical manipulation checks were met, with small detriments due to sequence effects.

### Group differences in changes in state measures from pre- to post-checking conditions

Table 2 shows the means and standard deviations of state measures by group, condition and time of measurement. In this section, we report inferential statistics only for effects that reached significance in the ANOVAs. Detailed statistical results are provided in S1 Table and significant interactions of selected state measures are illustrated in S2 Fig.

**Table 2:** Means and standard deviations of state measures.

	Group	Low-risk group (*n* = 76)				High-risk group (*n* = 103)			
	Condition	Increased BC		Typical BC		Increased BC		Typical BC	
	Time	*M*	*SD*	*M*	*SD*	*M*	*SD*	*M*	*SD*
Drive for Thinness [EDI-2]	Pre	1.48	0.39	1.53	0.44	4.19	0.90	4.09	0.89
	Post	1.60	0.50	1.50	0.44	4.44	0.92	4.18	0.91
Bulimia [EDI-2	Pre	1.20	0.21	1.21	0.21	2.24	0.99	2.18	0.96
	Post	1.21	0.24	1.21	0.27	2.29	1.06	2.20	0.98
Body Dissatisfaction [BISS]	Pre	3.03	0.88	3.14	0.96	5.93	1.33	5.85	1.30
	Post	3.20	1.17	3.12	1.02	6.41	1.64	6.04	1.36
Positive Affect [PANAS]	Pre	3.23	0.89	3.09	0.85	2.51	0.78	2.42	0.74
	Post	3.11	0.84	3.16	0.77	2.52	0.76	2.56	0.79
Negative Affect [PANAS]	Pre	1.55	0.52	1.53	0.50	2.21	0.82	2.16	0.72
	Post	1.57	0.49	1.59	0.49	2.42	0.89	2.22	0.79
General Pathology [PHQ-9]	Pre	0.36	0.35	0.41	0.35	0.95	0.59	0.94	0.52
	Post	0.43	0.35	0.39	0.34	1.00	0.56	0.94	0.53

As an overarching finding, the main effect of group was significant for all dependent variables and remained significant in all Bonferroni-adjusted post hoc tests (all *p*_adj_ < .001; drive for thinness: *F*(1, 177) = 673.49, *η*^2^_G_ = .763, bulimia: *F*(1, 177) = 87.17, *η*^2^_G_ = .301, body dissatisfaction: *F*(1, 177) = 320.58, *η*^2^_G_ = .573, positive affect: *F*(1, 177) = 37.09, *η*^2^_G_ = .140, negative affect: *F*(1, 177) = 57.36, *η*^2^_G_ = .197, general pathology: *F*(1, 177 = 78.31, *η*^2^_G_ = .256). The three-way interaction between group, condition, and time did not reach statistical significance for any of the examined dependent variables.

#### Drive for thinness

All two-way interactions were significant (group × condition: *F*(1, 177) = 6.52, *p* = .01, *η*^2^_G_ = .003, group × time: *F*(1, 177) = 7.88, *p* = .01, *η*^2^_G_ =. 002; time × condition: *F*(1, 177) = 13.00, *p* < .001, *η*^2^_G_ = .003). Following Bonferroni-adjusted post hoc tests, the simple main effect of condition failed to reach statistical significance for any combination. The simple main effect of time was significant for the increased BC condition in both the low-risk group (*p*_adj_ = .03) and the high-risk group (*p*_adj_ < .001).

#### Bulimia

None of the two-way interactions were significant (all *p* > .17).

#### Body dissatisfaction

All two-way interactions were statistically significant (group × condition: *F*(1, 177) = 4.09, *p* = .04, *η*^2^_G_ = .002; group × time: *F*(1, 177) = 5.72, *p* = .02, *η*^2^_G_ = .003; time × condition: *F*(1, 177) = 5.07, *p* = .03, *η*^2^_G_ = .002). In post hoc tests, the simple main effect of condition was not significant for any combination, and only the simple main effect of time remained significant in the increased BC condition in the high-risk group (*p*_adj_ < .001).

As *MC5* revealed a significant influence of sequence on body dissatisfaction, we conducted further analyses with split data sets: When the increased BC condition took place first (S1), the three-way interaction was significant (*F*(1, 77) = 4.64, *p* = .03, *η*^2^_G_ = .004). Post hoc tests confirmed the significance of the simple main effect of condition at post-measurement in the high-risk group (*p*_adj_ = .01). When the typical BC occurred first (S2), there were two significant two-way interactions (group × time: *F*(1, 98) = 7.26, *p* = .01, *η*^2^_G_ = .005; condition × time: *F*(1, 98) = 4.29, *p* = .04, *η*^2^_G_ = .004). In post hoc tests, the simple main effect of condition disappeared, and the simple main effect of time remained significant for the high-risk group in the typical (*p*_adj_ = .03) and the increased BC condition (*p*_adj_ = .01).

#### Positive affect

In the analysis of the entire data set, in which responses on the erroneously provided 6-point Likert scale had been corrected, only the two-way interaction between condition and time was significant (*F*(1, 177) = 6.46, *p* = .01, *η*^2^_G_ = .002). In post hoc tests, the simple main effect of condition was not significant for any combination, and the simple main effect of time was only significant for the typical BC condition in the high-risk group (*p*_adj_ = .04). An additional analysis of PA, with data captured using the correct 5-point Likert scale in cohorts 2 and 3 only, is depicted in S2 Table.

#### Negative affect

In the analysis of the entire data set, there was a significant two-way interaction between group and condition (*F*(1, 177) = 3.97, *p* = .049, *η*^2^_G_ = .002) and a significant main effect of time (*F*(1, 177) = 10.41, *p* = .001, *η*^2^_G_ = .004). In post hoc tests, the simple main effect of condition was no longer significant for any combination, and the simple main effect of time was only significant in the increased BC condition in the high-risk group (*p*_adj_ < .001).

#### General pathology

No significant two-way interactions emerged (all *p* > .06).

### Relationship of intolerance of uncertainty with BC and BC-induced changes

The results revealed no significant correlation between the inverted intolerance of uncertainty measure and the amount of BC at baseline in either the low-risk group (*S* = 72578, *p* = .95, *ρ* = .008) or the high risk-group (*S* = 181004, *p* = .95, *ρ* = .006). Correlations did not significantly differ between the groups (*z* = .62, *p* = .27, 95% CI [–.20, .39]).

A comparison of models drawing on one predictor (~group) or two predictors (~group+intolerance) showed that adding intolerance of uncertainty did not significantly improve the regression of change in the increased BC condition for bulimia (*F*(177, 176) = .10, *p* = .76), body dissatisfaction (*F*(177, 176) = .08, *p* = .78), positive affect (*F*(177, 176) = .04, *p* = .85), negative affect (*F*(177, 176) = 1.74, *p* = .19), or general pathology (*F*(177, 176) = .002, *p* = .97). The respective two-predictor models and regression coefficients of intolerance of uncertainty were not statistically significant. Only the change in drive for thinness was better predicted by the significant two-predictor model (*F*(2, 176) = 4.66, *p* = .01, *R*^2^_adj_ = .04 with *b*_group_ = .12, *β*_group_ = .28, *p*_group_ = .07 and *b*_intolerance_ = –.09, *β*_intolerance_ = –.15, *p*_intolerance_ = .04) than by the one-predictor model (*F*(1, 177) = 5.00, *p* = .03, *R*^2^_adj_ = .02 with *b*_group_ = .14, *β*_group_ = .33, *p*_group_ = .03), insofar as significantly more variance was explained (*F*(177, 176) = 4.22, *p* = .04, Δ*R^2^* = .023). However, this finding should be interpreted with caution, as the regression model assumptions were only partially met.

## Discussion

This experimental study aimed to investigate the theoretically proposed negative longer-term consequences of repeated BC [2, 12] in non-clinical women with low vs. high ED risk by manipulating BC frequency in their natural environment. The two groups were formed based on the extent of body concern, and showed similar demographic characteristics but also differed fundamentally from each other regarding their habitual amount of BC and in all of the constructs assessed. We hypothesized that BC would result in a deterioration of state measures in general (*H1*), and that these effects would be stronger in the increased BC condition compared to in the typical BC condition (*H2*) and stronger in the high-risk group than in the low-risk group (H3). While all threefold interactions were non-significant, distinguishable BC effects emerged. In summary, measures of psychopathology regarding bulimic and depressive symptoms did not alter in the three days of either typical or increased BC. However, increased BC led to a deterioration of body image-related symptoms (i.e., drive for thinness and body dissatisfaction) in the high-risk group. Moreover, the high-risk group showed significant changes of affect, namely a surprising increase in positive affect in the typical BC condition and the expected increase in negative affect in the increased BC condition. In the low-risk group, state measures were mostly unaffected, with the exception of a significant increase in drive for thinness when participants were required to increase their habitual amount of BC (i.e., in the increased BC condition). Our exploratory analysis of intolerance of uncertainty did not indicate a particularly influential role in BC-induced effects, which warrants further discussion.

### Body image-related symptomatology

In our study, we captured two core features of body image disturbances that have also been shown to predict the development of EDs [54]: First, drive for thinness is conceptualized as a cognitive bias that alters information processing and fosters typical ED behaviors (e.g., BC [12]). Three days of increased BC led to a significant increase in drive for thinness in both groups, in line with our theory-driven hypothesis of a negative impact of repeated BC. Contrary to expectation, this change did not significantly differ between the groups. While drive for thinness was more than twice as high in the high-risk group, the low-risk group also showed increased drive for thinness. The second core ED symptom assessed as part of a disturbed body image [55] was subjective body dissatisfaction, conceptually associated with drive for thinness [56], yet not identical and more evaluative in nature. Body dissatisfaction also rose significantly in the increased BC condition, but only for the high-risk group. Hence, increased BC frequency negatively affected women with high body concern and worsened their symptomology. As the typical BC frequency had no significant effects on either drive for thinness or body dissatisfaction, the habitual behavior merely seemed to maintain the status quo. The additional analyses of body dissatisfaction that were run with data sets split by sequence to counter the significant sequence effect in *MC5* revealed different results for the high-risk group depending on which BC condition was conducted first: When women with high body concern underwent the increased BC condition first and the typical BC condition second (S1), their extent of body dissatisfaction was significantly greater after increased BC. Yet, the overall change in body dissatisfaction over time, which we were interested in, did not differ between conditions. When women with high body concern underwent the typical BC condition first and the increased BC condition second (S2), their increase in body dissatisfaction became significant over time, but in both conditions. Since these divergent findings were based on small subsample sizes (i.e., high-risk group in S1: *n* = 48 and S2: *n* = 55), they lack statistical power and should not be considered reliable without further replication. Nevertheless, one might speculate that starting with a very inflated BC frequency and then reducing it to the previously habitual amount may mitigate the effects that become apparent in the comparatively more pleasant second condition of typical BC. Nonetheless, no significant sequence effects were observed in the other analyses.

Although correlational studies have reported significant associations between BC and ED symptoms in non-clinical samples (e.g., ED behaviors and cognitions [10]) and samples with high body concern (e.g., body dissatisfaction [18]), experimental studies on repeated BC have often failed to replicate these effects in the longer term: In the aforementioned study by Shafran et al. [20], in which non-clinical women critically checked their body in the mirror for 30 minutes, the reported increases in body dissatisfaction, feelings of fatness, and body-related self-critical thinking were only short-lived. Similarly, in the study by Bailey and Waller [21], the repeated checking of one’s wrist size for eight hours did not alter body dissatisfaction or eating attitudes in non-clinical women, and Opladen et al. [19] likewise did not find an increase in overall ED symptoms. By contrast, our results, obtained using the same study design as Opladen et al. [19], demonstrated specific effects on both constructs in the increased BC condition, namely, a worsening of body image-related symptomology. However, because the effects of the condition dissolved in post hoc testing, we cannot infer a strong influence of BC frequency in our study. As a potential explanation for this finding, it can be assumed that information provided to the participants about BC increased their body-focused attention, thus creating effects from the outset and also impacting the state measures in the typical BC condition. An attentional bias may likewise apply to the significant effects found in the increased condition, which may partly or entirely reflect a change in reporting behavior due to densely presented questionnaires with a noticeable focus on body image and ED pathology. Additionally, the two conditions might have been less distinct than intended, since the requested threefold increase in BC was not completely realized overall, with some participants remarking that tripling their already high habitual BC baseline was simply not achievable in everyday life. Possibly, however, participants may have had an intuitive perception of what is good for them and a healthy resistance against “too much” BC behavior, which may also have impacted the findings. This personal notion of an “innocuous” BC limit, and the risks inherent in exceeding it, might represent a crucial difference between non-clinical individuals and ED patients, illuminating why BC does not cause damage by default [19].

### Affect

Theories assume that the driving force behind negative consequences of BC is a deterioration of affect over time, which amplifies the need for regulation [2, 12]. Our findings only partly reflect this assumption: In the high-risk group, negative affect increased significantly in the increased BC condition, which corresponds to correlation studies in samples with comparable levels of body concern (e.g. [16, 17]). In the typical BC condition, however, their negative affect did not significantly increase, which might indicate emotional adaptation, i.e., a decreased emotional reaction to habitually performed BC over time. Following theoretical assumptions by Williamson et al. [12] and considering the results of the high-risk group altogether, one could speculate that the actual risk of BC might stem from an increase in its frequency. Since BC with the typical frequency did not increase negative affect, drive for thinness, or body dissatisfaction, this does not seem to drive a BC increase as theoretically postulated. At least, this impression emerged from our data on relatively short examination durations. So far, only one longitudinal study has focused on long-term consequences of habitually performed BC [9], and found that BC at baseline significantly predicted increases in ED pathology for male and female adolescents four months later. The reverse relationship of ED pathology at baseline predicting changes in BC only pertained to males. The authors in line with others (e.g., [7, 57]) concluded that “if supported by further research, targeting BC behaviors in prevention programs may be warranted” [9]. On the basis of our findings (i.e., deteriorations when vulnerable women increased their BC), we likewise encourage research using longer time frames and employing a design that is sensitive to potentially very gradual long-term changes in state measures through habitual BC. If this indeed influences the perceived need for and the amount of BC, the potential harm should be addressed as early as possible, when vulnerability exists but full-blown ED pathology might still be prevented, e.g., through psychoeducation and imparting constructive emotion regulation strategies. Knowledge and awareness could perhaps strengthen individuals’ perception of what constitutes an innocuous amount of BC, and prevent them from gradually exceeding this amount.

In the low-risk group, levels of negative affect remained unchanged, independent of condition, as was the case for body dissatisfaction, in contrast to Opladen et al. [19], who found a general increase in negative affect in both conditions using the same design in community women. We believe that there are two main reasons for this discrepancy: First, the examined samples differ considerably between the two studies, as Opladen et al. [19] did not screen for the extreme ends of body concern (i.e., almost no or very high body concern in the present study). As the mean baseline values of their sample (e.g., the habitual BC amount) lay approximately in between those of our two subsamples, their findings may have bundled different effects of BC in people with diverging body concern. Second, fundamental theories on BC only refer to vulnerable individuals with a highly developed negative body self-schema [12] and do not make statements about healthy populations. Since neither typical nor increased BC led to a deterioration of negative affect or body dissatisfaction in women with low body concern in the present study, this might suggest that BC merely imparts neutral body-related information to these women, especially if they pay less attention to disliked body parts, as has been found in previous studies (e.g., [17]). Nevertheless, the requested increase in BC did enhance their preoccupation with thinness, possibly because it prompted a rather positive approach motivation for eating regulation (i.e., an autonomous focus on health and well-being; [58]), which is less associated with ED development. In women with high body concern, by contrast, drive for thinness might rather have been based on maladaptive avoidance motivation to counteract negative assumptions and fears regarding body weight, shape, or size (i.e., a controlled focus on appearance; [58]), as indicated in previous research (e.g., [3]).

Along with negative emotional experience, we further captured positive affect, which has often been neglected in BC research. Surprisingly, the high-risk group showed a significant increase in positive affect in the typical BC condition, which was the opposite direction of effect than expected. Thus, habitual behavior did not change negative affect, but did induce feelings like strength, inspiration, or determination in women with high body concern. So far, an “improvement” of affect has only been theorized as occurring through a reduction of negative affect, as a direct short-term effect of BC. Our longer-term findings now open up a new perspective: Increased positive affect might support the maintenance of BC, possibly due to the pleasant attainment of certainty, motivation, or control, which were found to be the most prominently endorsed functions of BC in the study by Opladen et al. [19]. When BC was increased, the affect-enhancing effect disappeared in the high-risk group, and the low-risk group showed a slight but non-significant decrease in positive affect, which again suggests that they exceeded a limit of what might be deemed an innocuous amount of BC. In sum, changes in positive affect might play a less important role in the development of negative consequences of BC than vice versa.

### Psychopathology

For the Bulimia subscale of the EDI-2 and the PHQ-9, we found no significant effects, except for the evident difference in burden between the groups. Hence, regardless of ED risk and BC frequency, neither bulimic symptom severity (e.g., secret or uncontrollable eating) nor general pathology (e.g., core depressive symptoms, sleep disturbances, suicidality) changed over the course of three days. In contrast to the other measures applied, these two constitute core symptoms of mental disorders which develop more slowly, partially unfold on a behavioral level, and most likely cannot be changed through a relatively brief manipulation. Nevertheless, given that the experimental increase in BC led to a deterioration of cognitive-evaluative-emotional characteristics that promote EDs in at-risk women, we also conjecture that psychopathology may subsequently increase, such that clinical thresholds might be passed in the long term. This assumption is supported by BC-based predictions of bulimic binging and purging [10] as well as mental and physical health impairments [15]. Moreover, Opladen et al. [19] found significant changes in PHQ-9 scores, i.e., an improvement in the typical BC condition and a deterioration in the increased BC condition. We were unable to replicate these results, which may be attributable to the aforementioned disparity between the samples. The difference in means before and after BC indicated similar directions of effects (i.e., less general pathology in the typical BC condition in the low-risk group, and more general pathology in the increased BC condition in both groups), but these changes were non-significant. If Opladen`s findings were to be confirmed with statistical significance, the question would arise of whether, when, and for whom BC may possibly exert a positive impact [19].

### Intolerance of uncertainty

Although the multifactorial development of EDs has been extensively researched, causal associations and interactions with influencing factors are still to be uncovered [59]. Therefore, from an exploratory perspective, we assessed intolerance of uncertainty as a potential risk factor in association with longer-term consequences of BC. Indeed, the high-risk group exhibited greater intolerance of uncertainty (i.e., less tolerance) in the basic survey. In contrast to *H4*, there was no association with the habitual amount of BC, not even in women with high body concern. Moreover, the consideration of intolerance of uncertainty mostly did not improve the prediction of change beyond that by group in *H5*. We only found intolerance of uncertainty to play a significant role in the BC-driven increase in drive for thinness. Retrospectively, we believe that our choice of questionnaire essentially prevented us from detecting significant effects due to its unspecific items (e.g., “I like it when work runs smoothly.”), lacking validity in the field of mental disorders. Furthermore, the UGTS targets tolerance rather than intolerance of uncertainty, which do not necessarily represent opposite poles of the same construct. Instead, we would recommend using the twelve-item Intolerance of Uncertainty Scale (IUS-12; [60]), which has shown good psychometric properties in ED populations [25]. The IUS-12 has previously been used in a study by Bijsterbosch et al. [26], which is the only study to date to have investigated the relations between IU and BC in individuals with EDs. Additionally, future studies should assess BC motivations similarly to Kachani et al. [5] and Opladen et al. [19], paying special attention to participants’ extent of intolerance of uncertainty, their intention to increase certainty, and whether BC successfully meets this desire in the short and/or long(er) term.

### Limitations, strengths and outlook

When evaluating the findings of the present study, several aspects need to be considered: As with online studies in general, self-selection could have biased our final sample, although, this risk is mitigated by the fact that only about one third of applicants were included in the study due to our strict screening criteria for body concern. We successfully established two very different subsamples, with the high-risk group habitually engaging in BC more frequently, as is known from other studies [7, 20], and scoring significantly worse on all dependent variable measures irrespective of our BC manipulation. Therefore, these women, undiagnosed and untreated at the time of the study, carried a considerable mental health burden and were truly at risk for a mental disorder (e.g., an ED). Aiming to improve Opladen et al.’s [19] data collection, we provided participants with a large amount of information from the outset, repeatedly sent reminders for precise recording, and communicated in the style of a supportive friend in order to promote honesty and motivation. This approach aimed to ensure that instructions were properly followed despite the limited control inherent to our anonymized online design. Nevertheless, we can assume that our naturalistically gathered data are subject to confounding variables related to participants’ lifestyles (e.g., social interactions impacting responses). To ensure valid results, a considerable number of participants were excluded due to a failure to complete all of the surveys, exceeding the time restrictions, or not sufficiently increasing their habitual BC. The time frame that participants should refer to when answering our state measures was adapted from established self-report instruments for the present study, and instructions were rephrased accordingly. This was necessary due to the lack of questionnaires with the time frame focused on. However, it means some measures (i.e., EDI-2 and PHQ-9) have not been validated for the chosen time frame, e.g., regarding their sensitivity to change, which should be taken into account when interpreting our findings. In statistical terms, the two significant sequence biases of the manipulation checks should be noted, although an influence on the BC increase factor, with a small effect size, seems unproblematic because this was not part of our analysis (see *MC3*). However, the body dissatisfaction results need to be interpreted with caution (see *MC5*), as was indeed the case in the present study. Due to the number of statistical tests conducted, the increased risk of type I errors must be kept in mind, which we counteracted through the use of a conservative correction method. The risk of type II errors remains because our sample size was designed to detect medium effects, meaning we may have missed significant small effects, such as three-way interactions.

One important strength of our study is its naturalistic approach, which took interindividual differences in BC frequency into account and allowed participants to stick to their habitual strategies (e.g. only mirror checking) embedded in their everyday life. Certainly, differences remain between natural BC behavior, which may increase over time, and our experimental setup, where we first needed to bring a predominantly unaware everyday behavior to our participants’ awareness [61] and later called for an artificial BC increase (which, importantly, was not fully implemented). As previously discussed, our results might also reflect less psychologically meaningful changes in reporting behavior rather than genuine changes in affect or cognitions. However, our goal was not to specifically alter state measures but rather to experimentally evaluate whether increased BC would affect women with no current diagnosis of a mental disorder but with varying levels of ED risk. The generalizability of the findings is limited to late adolescents and young adult female populations, although this is an important time frame as it represents the peak onset period of EDs [62, 63].

Future replications of our study could extend the focus to different samples (e.g., exclusively examining male participants or clinical samples). For a better empirical understanding of the interplay of the impact of repeated BC on body image disturbances and ED development with other factors, many additional constructs could be assessed, such as weight bias internalization [64, 65]. Beyond our focus on BC frequency, analyzing other features of BC that are known to differ between clinical and healthy populations, as well as across specific disorders, would also be relevant. For instance, the length of a BC episode (typically < 2 minutes in EDs according to [61]) may influence whether repeated BC is harmful or beneficial in the long(er) term, with longer episodes possibly resulting in either habituation or inhibitory learning [22]. Additionally, study findings differ depending on whether the focus is on liked versus disliked body parts (e.g., [22]) or on examining the body as a whole (e.g., [66]), which is a crucial consideration for body exposure therapy [67].

### Conclusions

Recently, the trend toward increasing levels of body dissatisfaction among adolescents of both sexes [68] has been highlighted as a “global mental health concern”, and greater efforts are required in order to prevent or reduce this [69]. Therefore, we investigated the longer-term consequences of a prominent ED-associated behavior in women who are especially vulnerable due to high body concern. Adding to the only small body of experimental knowledge, our online study revealed that habitual BC repeatedly conducted in participants’ natural environment exerted no negative impact. Contrary to theoretical postulates, significant deteriorations only emerged when BC was increased, and particularly affected vulnerable women who had high body concern in terms of drive for thinness, body dissatisfaction, and negative affect. The bulimic and depressive psychopathology of participants remained unaffected, but might have deteriorated with a longer duration of the study. Women who were content with their bodies seemed to intuitively preserve a lower, harmless amount of BC, and increasing this amount only strengthened their cognitive preoccupation with thinness, the repercussions of which remain unknown.

## Supporting information

S1 Fig Participant flow and data cleaning process. Randomized sequence order: S1 (increased BC condition first) or S2 (typical BC condition first).
(DOCX)

S2 Fig Graphical illustrations of significant interactions on state measures of drive for thinness (a), body dissatisfaction (b), and negative affect (c). Linear graphs for both BC conditions (black line/triangle: typical BC condition, grey line/dot: increased BC condition) split by group (left: low-risk group, right: high-risk group), x-axis: time of measurement (pre and post), y-axis: mean values of state measures with error bars of +/- one standard deviation.
(DOCX)

S1 Appendix References of R packages used. Presented in alphabetical order of package names.
(DOCX)

S1 Table Results of three-way analyses of variance. Body dissatisfaction was influenced by a significant sequence effect, as seen in *MC5*, and we therefore additionally analyzed split data sets for S1 (increased BC condition first) and S2 (typical BC condition first).
(DOCX)

S2 Table Additional results of three-way analyses of variance in narrower PA sample. Data of only *n* = 79 participants of cohorts 2 and 3.
(DOCX)

S1 Dataset (CSV)
