# Supplementary material for: Longer-term consequences of increased body checking in women at risk for eating disorders–a naturalistic experimental online study
Source: PLoS One. 2024 Dec 26;19(12):e0316190. doi: 10.1371/journal.pone.0316190 (PMC11671019; doi:10.1371/journal.pone.0316190)
Supplement: S1 Fig — Randomized sequence order: S1 (increased BC condition first) or S2 (typical BC condition first). (DOCX) [file pone.0316190.s001.docx]

**S1 Fig. Participant flow and data cleaning process.**





Randomized sequence order: S1 (increased BC condition first) or S2 (typical BC condition first).
