# Supplementary material for: Longer-term consequences of increased body checking in women at risk for eating disorders–a naturalistic experimental online study
Source: PLoS One. 2024 Dec 26;19(12):e0316190. doi: 10.1371/journal.pone.0316190 (PMC11671019; doi:10.1371/journal.pone.0316190)
Supplement: S2 Fig — Graphical illustrations of significant interactions on state measures of drive for thinness (a), body dissatisfaction (b), and negative affect (c). Linear graphs for both BC conditions (black line/triangle: typical BC condition, grey line/dot: increased BC condition) split by group (left: low-risk group, right: high-risk group), x-axis: time of measurement (pre and post), y-axis: mean values of state measures with error bars of +/- one standard deviation. (DOCX) [file pone.0316190.s002.docx]

**S2 Fig. Graphical illustrations of significant interactions on state measures of drive for thinness (a), body dissatisfaction (b), and negative affect (c).**

| (a) | 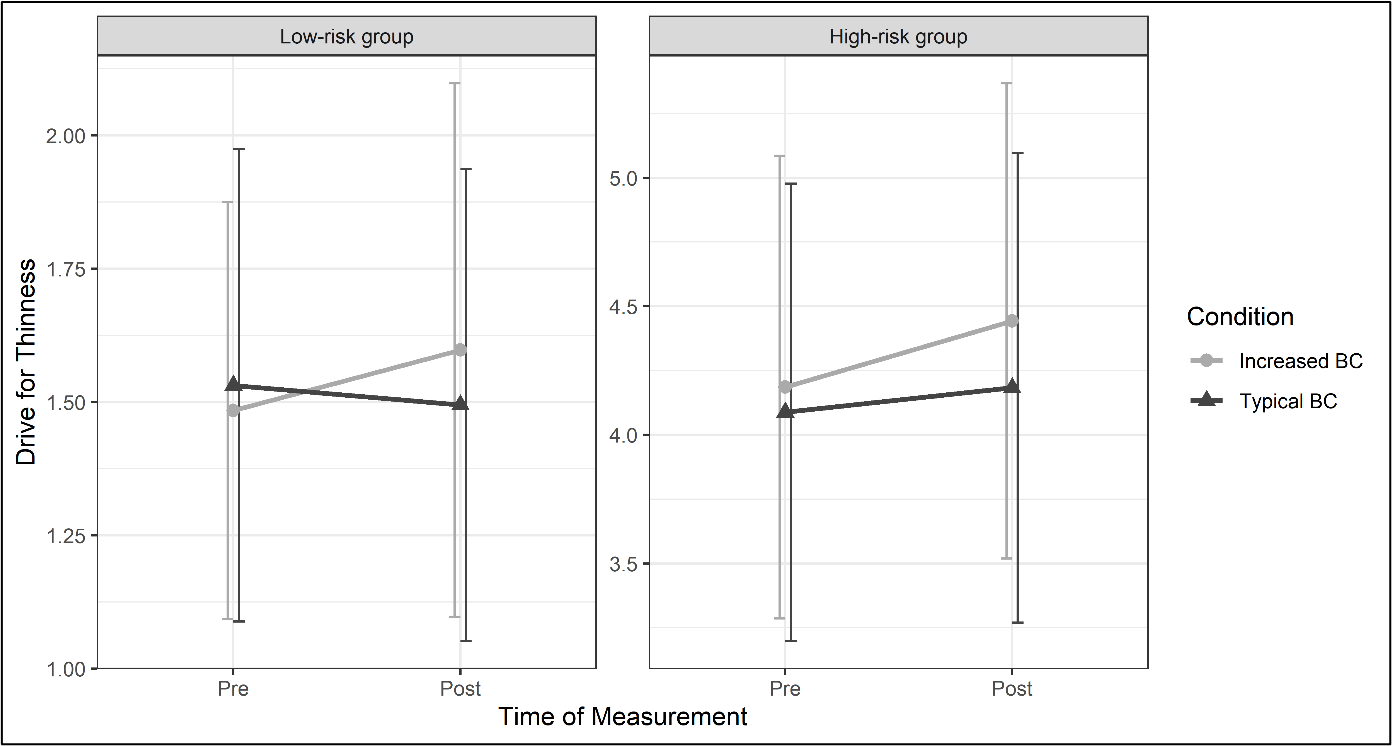 |
| --- | --- |
| (b) | 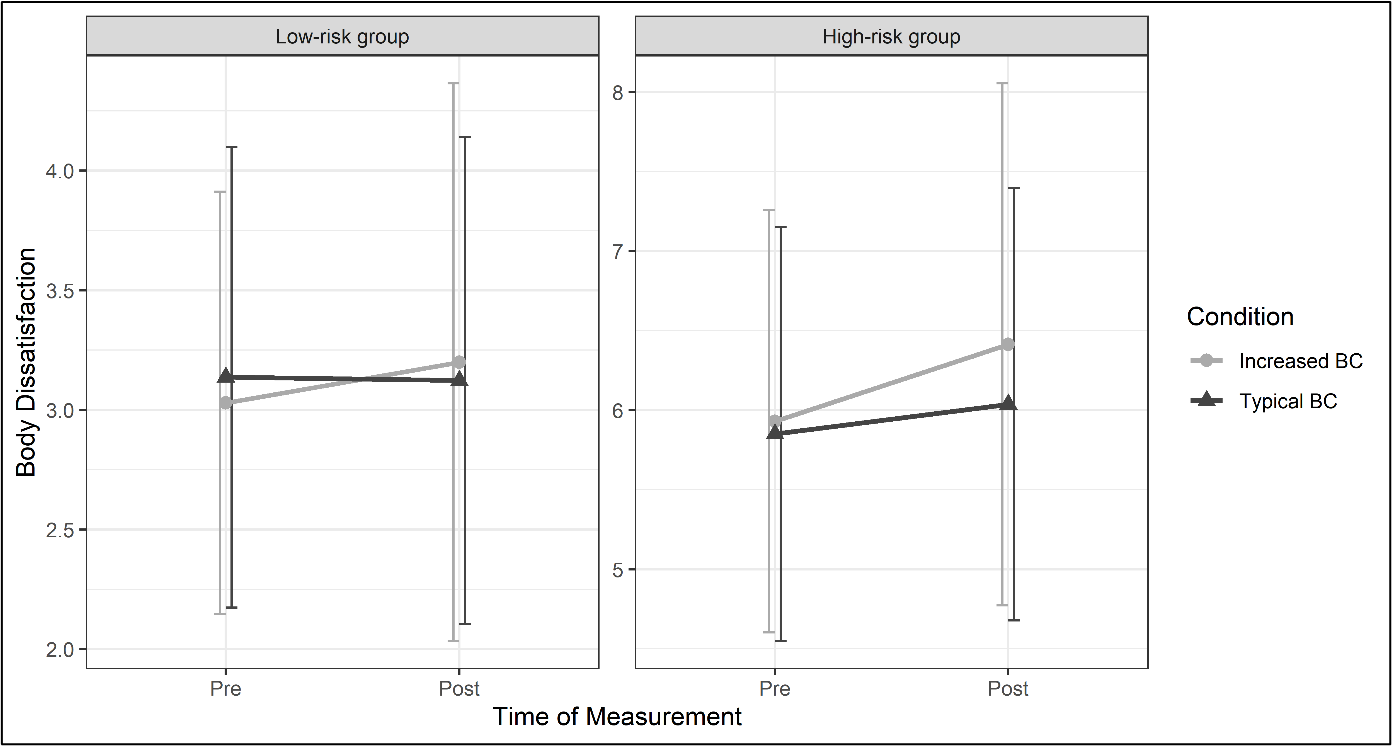 |
| (c) | 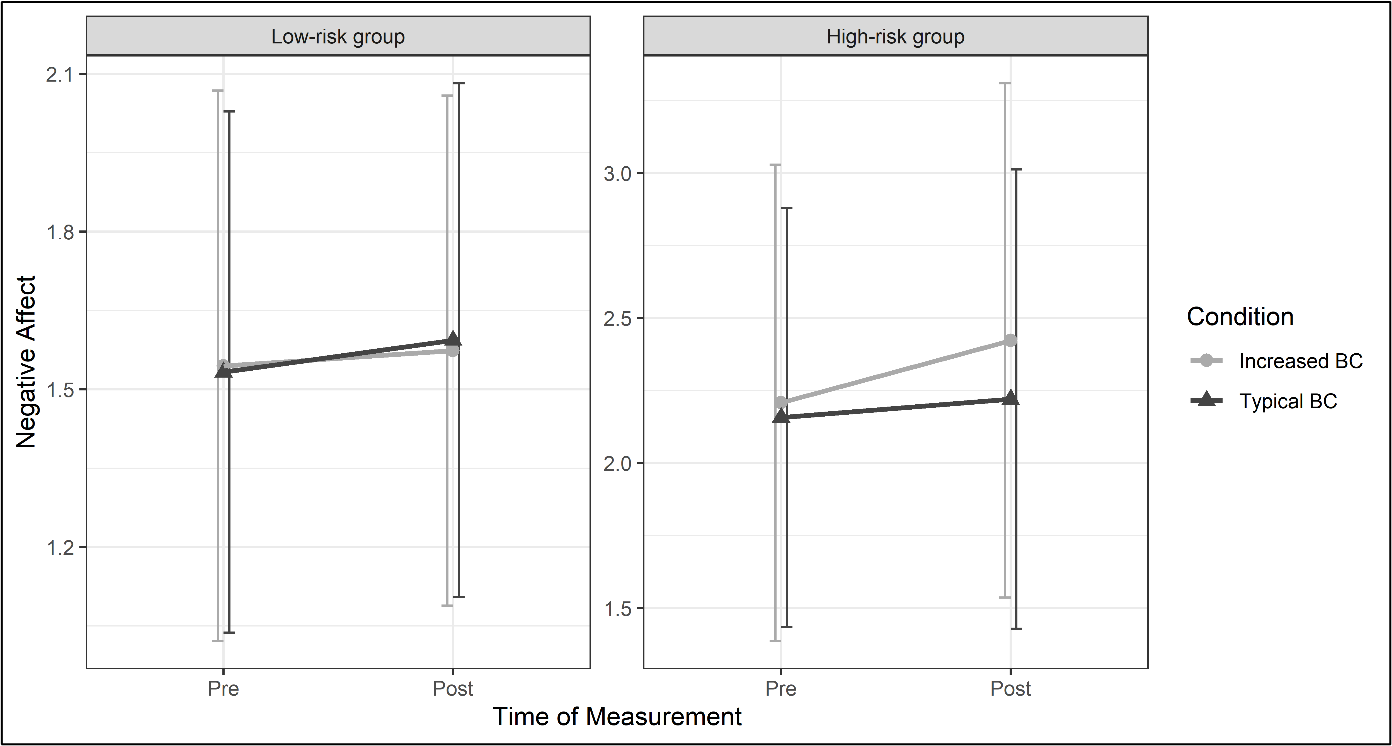 |

Linear graphs for both BC conditions (black line/triangle: typical BC condition, grey line/dot: increased BC condition) split by group (left: low-risk group, right: high-risk group), x-axis: time of measurement (pre and post), y-axis: mean values of state measures with error bars of +/- one standard deviation.
