# Supplementary material for: Longer-term consequences of increased body checking in women at risk for eating disorders–a naturalistic experimental online study
Source: PLoS One. 2024 Dec 26;19(12):e0316190. doi: 10.1371/journal.pone.0316190 (PMC11671019; doi:10.1371/journal.pone.0316190)
Supplement: S1 Appendix — Presented in alphabetical order of package names. (DOCX) [file pone.0316190.s003.docx]

**S1 Appendix. References of R packages used.** Presented in alphabetical order of package names.

| apaTables | Stanley D. apaTables: Create American Psychological Association (APA) Style Tables; 2021. Available from: URL: https://CRAN.R-project.org/package=apaTables. |
| --- | --- |
| base | R Core Team. R: A Language and Environment for Statistical Computing. Vienna, Austria; 2023. Available from: URL: https://www.R-project.org/. |
| boot | Canty A, Ripley B. boot: Bootstrap R (S-Plus) Functions; 2021. |
| car | Fox J, Weisberg S. An R Companion to Applied Regression. Third. Thousand Oaks CA: Sage 2019. |
| dplyr | Wickham H, François R, Henry L, Müller K. dplyr: A Grammar of Data Manipulation; 2022. Available from: URL: https://CRAN.R-project.org/package=dplyr. |
| ggplot2 | Wickham H. ggplot2: Elegant Graphics for Data Analysis. Springer-Verlag New York 2016. |
| ggpubr | Kassambara A. ggpubr: ‘ggplot2’ Based Publication Ready Plots; 2020. Available from: URL: https://CRAN.R-project.org/package=ggpubr. |
| graphics | R Core Team. R: A Language and Environment for Statistical Computing. Vienna, Austria; 2023. Available from: URL: https://www.R-project.org/. |
| MASS | Venables WN, Ripley BD. Modern Applied Statistics with S. Fourth. New York: Springer 2002. |
| Matrix | Bates D, Maechler M, Jagan M. Matrix: Sparse and Dense Matrix Classes and Methods; 2022. Available from: URL: https://CRAN.R-project.org/package=Matrix. |
| lavaan | Rosseel Y. lavaan : An R Package for Structural Equation Modeling. J. Stat. Soft. 2012; 48(2) [https://doi.org/10.18637/jss.v048.i02] |
| lsr | Navarro D. Learning statistics with R: A tutorial for psychology students and other beginners. (Version 0.6). Sydney, Australia 2015. |
| plyr | Wickham H. The Split-Apply-Combine Strategy for Data Analysis. J. Stat. Soft. 2011; 40(1) [https://doi.org/10.18637/jss.v040.i01] |
| psych | Revelle W. psych: Procedures for Psychological, Psychometric, and Personality Research: Northwestern University; 2022. Available from: URL: https://CRAN.R-project.org/package=psych. |
| QuantPsyc | Fletcher TD. QuantPsyc: Quantitative Psychology Tools; 2022. Available from: URL: https://CRAN.R-project.org/package=QuantPsyc. |
| reshape | Wickham H. Reshaping Data with the reshape Package. J. Stat. Soft. 2007; 21(12) [https://doi.org/10.18637/jss.v021.i12] |
| rstatix | Kassambara A. rstatix: Pipe-Friendly Framework for Basic Statistical Tests; 2021. Available from: URL: https://CRAN.R-project.org/package=rstatix. |
| stats | R Core Team. R: A Language and Environment for Statistical Computing. Vienna, Austria; 2023. Available from: URL: https://www.R-project.org/. |
| utils | R Core Team. R: A Language and Environment for Statistical Computing. Vienna, Austria; 2023. Available from: URL: https://www.R-project.org/. |
