# Supplementary material for: Longer-term consequences of increased body checking in women at risk for eating disorders–a naturalistic experimental online study
Source: PLoS One. 2024 Dec 26;19(12):e0316190. doi: 10.1371/journal.pone.0316190 (PMC11671019; doi:10.1371/journal.pone.0316190)
Supplement: S1 Table — Body dissatisfaction was influenced by a significant sequence effect, as seen in MC5, and we therefore additionally analyzed split data sets for S1 (increased BC condition first) and S2 (typical BC condition first). (DOCX) [file pone.0316190.s004.docx]

**S1 Table. Results of Three-Way Analyses of Variance.** Body dissatisfaction was influenced by a significant sequence effect, as seen in *MC5*, and we therefore additionally analyzed split data sets for S1 (increased BC condition first) and S2 (typical BC condition first).

| Variable | Effect | *F*(1,177) | *p* | | *η*²_G_ |
| --- | --- | --- | --- | --- | --- |
| Drive for Thinness | **Group** | **673.49** | **3.14E–62** | ***** | **0.763** |
| [EDI-2] | **Condition** | **12.19** | **6.07 E–04** | ***** | **0.005** |
|  | **Time** | **19.11** | **2.10 E–05** | ***** | **0.005** |
|  | **Group × Condition** | **6.52** | **0.012** | ***** | **0.003** |
|  | **Group × Time** | **7.88** | **0.006** | ***** | **0.002** |
|  | **Condition × Time** | **13.00** | **4.05 E–04** | ***** | **0.003** |
|  | Group × Condition × Time | 0.02 | 0.892 |  | < 0.001 |
| Bulimia | **Group** | **87.17** | **4.19 E–17** | ***** | **0.301** |
| [EDI-2] | Condition | 1.71 | 0.193 |  | < 0.001 |
|  | Time | 0.79 | 0.377 |  | < 0.001 |
|  | Group × Condition | 1.88 | 0.173 |  | < 0.001 |
|  | Group × Time | 0.37 | 0.545 |  | < 0.001 |
|  | Condition × Time | 0.37 | 0.546 |  | < 0.001 |
|  | Group × Condition × Time | 0.28 | 0.600 |  | < 0.001 |
| Body Dissatisfaction | **Group** | **320.58** | **1.39 E–41** | ***** | **0.573** |
| [BISS] | Condition | 3.10 | 0.080 |  | 0.002 |
|  | **Time** | **14.88** | **1.61 E–04** | ***** | **0.007** |
|  | **Group × Condition** | **4.09** | **0.045** | ***** | **0.002** |
|  | **Group × Time** | **5.72** | **0.018** | ***** | **0.003** |
|  | **Condition × Time** | **5.07** | **0.026** | ***** | **0.002** |
|  | Group × Condition × Time | 0.28 | 0.600 |  | < 0.001 |
| Positive Affect | **Group** | **37.09** | **6.84 E–09** | ***** | **0.140** |
| [PANAS] | Condition | 0.90 | 0.343 |  | < 0.001 |
|  | Time | 0.76 | 0.384 |  | < 0.001 |
|  | Group × Condition | 0.09 | 0.760 |  | < 0.001 |
|  | Group × Time | 2.63 | 0.107 |  | < 0.001 |
|  | Condition × Time | 6.46 | 0.012 | * | 0.002 |
|  | Group × Condition × Time | 0.17 | 0.682 |  | < 0.001 |
| Negative Affect | **Group** | **57.36** | **1.94 E–12** | ***** | **0.197** |
| [PANAS] | Condition | 3.50 | 0.063 |  | 0.002 |
|  | **Time** | **10.41** | **0.001** | ***** | **0.004** |
|  | **Group × Condition** | **3.97** | **0.048** | ***** | **0.002** |
|  | Group × Time | 2.74 | 0.100 |  | 0.001 |
|  | Condition × Time | 1.13 | 0.290 |  | < 0.001 |
|  | Group × Condition × Time | 2.63 | 0.107 |  | 0.001 |
| General Pathology | **Group** | **78.31** | **8.9 E–16** | ***** | **0.256** |
| [PHQ-9] | Condition | 0.32 | 0.574 |  | < 0.001 |
|  | Time | 1.77 | 0.185 |  | < 0.001 |
|  | Group × Condition | 1.21 | 0.273 |  | < 0.001 |
|  | Group × Time | 0.02 | 0.880 |  | < 0.001 |
|  | Condition × Time | 3.65 | 0.058 |  | 0.001 |
|  | Group × Condition × Time | 0.40 | 0.527 |  | < 0.001 |

| Variable | Effect | *F*(1,77) | *p* | | *η*²_G_ |
| --- | --- | --- | --- | --- | --- |
| Body Dissatisfaction, | **Group** | **199.06** | **4.76 E–23** | ***** | **0.648** |
| only S1 [BISS] | Condition | 2.17 | 0.145 |  | 0.004 |
|  | Time | 2.79 | 0.099 |  | 0.003 |
|  | **Group × Condition** | **5.32** | **0.024** | ***** | **0.009** |
|  | Group × Time | 0.40 | 0.528 |  | < 0.001 |
|  | Condition × Time | 0.62 | 0.434 |  | < 0.001 |
|  | **Group × Condition × Time** | **4.64** | **0.034** | ***** | **0.004** |
| Variable | Effect | *F*(1,98) | *p* | | *η*²_G_ |
| Body Dissatisfaction, | **Group** | **139.67** | **1.46 E–20** | ***** | **0.52** |
| only S2 [BISS] | Condition | 0.89 | 0.348 |  | < 0.001 |
|  | **Time** | **14.06** | **2.99 E–04** | ***** | **0.01** |
|  | Group × Condition | 0.39 | 0.535 |  | < 0.001 |
|  | **Group × Time** | **7.26** | **0.008** | ***** | **0.005** |
|  | **Condition × Time** | **4.29** | **0.041** | ***** | **0.004** |
|  | Group × Condition × Time | 0.64 | 0.426 |  | < 0.001 |
