# Supplementary material for: Longer-term consequences of increased body checking in women at risk for eating disorders–a naturalistic experimental online study
Source: PLoS One. 2024 Dec 26;19(12):e0316190. doi: 10.1371/journal.pone.0316190 (PMC11671019; doi:10.1371/journal.pone.0316190)
Supplement: S2 Table — Data of only n = 79 participants of cohorts 2 and 3. (DOCX) [file pone.0316190.s005.docx]

**S2 Table. Additional results of three-way analyses of variance in narrower PA sample.** Data of only *n* = 77 participants from cohorts 2 and 3.

| Variable | Effect | *F*(1,76) | *p* | | *η*²_G_ |
| --- | --- | --- | --- | --- | --- |
| Positive Affect,  correctly captured with the 5-point Likert scale [PANAS] | **Group** | **12.788** | **6.11 E–04** | ***** | **0.111** |
|  | Condition | 0.049 | 0.825 |  | < 0.001 |
|  | Time | 3.362 | 0.071 |  | 0.004 |
|  | Group × Condition | 0.148 | 0.701 |  | < 0.001 |
|  | Group × Time | 0.134 | 0.716 |  | < 0.001 |
|  | Condition × Time | 0.204 | 0.653 |  | < 0.001 |
|  | Group × Condition × Time | 0.504 | 0.48 |  | < 0.001 |
|  | Group × Condition × Time | 1.01 | 0.318 |  | < 0.001 |
